# Nature Makes Glass

**Published:** 1892-11

**Authors:** 


### NATURE MAKES GLASS.

Did you ever see the diameter of a lightning flash measured? asked a geologist. Well, here is the case which once inclosed a flash of lightning, fitting it exactly, so that you can just see how big it was. This is called a "fulgurite," or "lightning hole," and the material it is made of is glass. I will tell you how it was manufactured, though it took only a fraction of a second to turn it out.

When a bolt of lightning strikes a bed of sand it plunges downward into the sand for a distance, less or greater, transforming simultaneously into glass the silica in the material through which it passes. Thus, by its great heat, it forms at once a glass tube of precisely its own size.

Now and then such a tube, known as a "fulgurite," is found and dug up. Fulgurites have been followed into the sand by excavations for nearly thirty feet. They vary in interior diameter from the size of a quill to three inches or more, according to the bore of the flash.

But fulgurites are not alone produced in sand; they are found also in solid rocks, though very naturally of slight depth and frequently existing merely as a thin glassy coating on the surface. Such fulgurites occur in astonishing abundance on the summit of Little Ararat, in Armenia.

The rock is soft and so porous that a block a foot long can be obtained perforated in all directions by little tubes filled with bottle green glass formed from the fused rock. There is a small specimen in the National Museum which has the appearance of having been bored by the toredo, the holes made by the worm subsequently filled with glass. I am indebted to the "Washington Star" for the foregoing accounts.

I may add that Charles Darwin mentions these fulgurites in his book of travels and Humboldt found some on the high Nevada de Zolnca, in Mexico. Humboldt ascended this precipitous peak at the risk of his own life.

